# Functional FDG-PET: Measurement of Task Related Neural Activity in Humans—A Compartment Model Approach and Comparison to fMRI

**DOI:** 10.3390/diagnostics13193121

**Published:** 2023-10-04

**Authors:** Saga Steinmann Madsen, Svend Hvidsten, Thomas Lund Andersen

**Affiliations:** 1 Center for Neuropsychiatric Depression (CNDR), Mental Health Center Glostrup, Capital Region of Denmark, 2600 Glostrup, Denmark; 2 Department of Clinical Research, Faculty of Health Sciences, University of Southern Denmark, 5000 Odense, Denmark; 3 Department of Nuclear Medicine, Odense University Hospital (OUH), 5000 Odense, Denmark; svend.hvidsten@rsyd.dk; 4 Department of Clinical Physiology & Nuclear Medicine, Rigshospitalet, 2300 København Ø, Denmark; thomas.lund.andersen@regionh.dk

**Keywords:** mental health, neuroimaging, functional FDG-PET, fMRI, glucose utilization, quantitative kinetics

## Abstract

Neuroimaging holds an essential position in global healthcare, as brain-related disorders are a substantial and growing burden. Non-degenerative disorders such as stress, depression and anxiety share common function related traits of diffuse and fluctuating changes, such as change in brain-based functions of mood, behavior and cognitive abilities, where underlying physiological mechanism remain unresolved. In this study we developed a novel application for studying intra-subject task-activated brain function by the quantitative physiological measurement of the change in glucose metabolism in a single scan setup. Data were acquired on a PET/MR-scanner. We implemented a functional [^18^F]-FDG PET-scan with double boli-tracer administration and finger-tapping activation, as proof-of-concept, in five healthy participants. The [^18^F]-FDG data were analyzed using a two-tissue compartment double boli kinetic model with an image-derived input function. For stand-alone visual reference, blood oxygenation level dependent (BOLD) functional MRI (fMRI) was acquired in the same session and analyzed separately. We were able to measure the cerebral glucose metabolic rate during baseline as well as activation. Results showed increased glucose metabolic rate during activation by 36.3–87.9% mean 62.0%, locally in the peak seed region of M1 in the brain, on an intra-subject level, as well as very good spatial accuracy on group level, and localization compared to the BOLD fMRI result at subject and group level. Our novel method successfully determined the relative increase in the cerebral metabolic rate of glucose on a voxel level with good visual association to fMRI at the subject-level, holding promise for future individual clinical application. This approach will be easily adapted in future clinical perspectives and pharmacological interventions studies.

## 1. Introduction

### 1.1. Background

Mental health disorders are rapidly becoming the most expensive burden of diseases in many cultures across the world [1]. Brain disorders are the leading cause of years lived with disability worldwide [2]. Neurodegenerative brain diseases involve the progressive deterioration of nervous system structure and function, whereas non-degenerative disorders such as, but not exclusive to, affective disorders like stress, depression, and anxiety do not manifest immediate structural or anatomical abnormalities that can be directly measured through neurobiological imaging methods. Not in any pathological or physiological ways, which we have not yet been able to measure directly from imaging in a neurobiological perspective, let alone to quantify on an intra-subject level. To give insight into disease pathology and understand the underlying mechanism neurobiology of mental health disorders and pharmacological intervention treatments, functional and dynamic changes during scanning may prove important [3].

### 1.2. Functional Brain Scans

**Figure 1:**
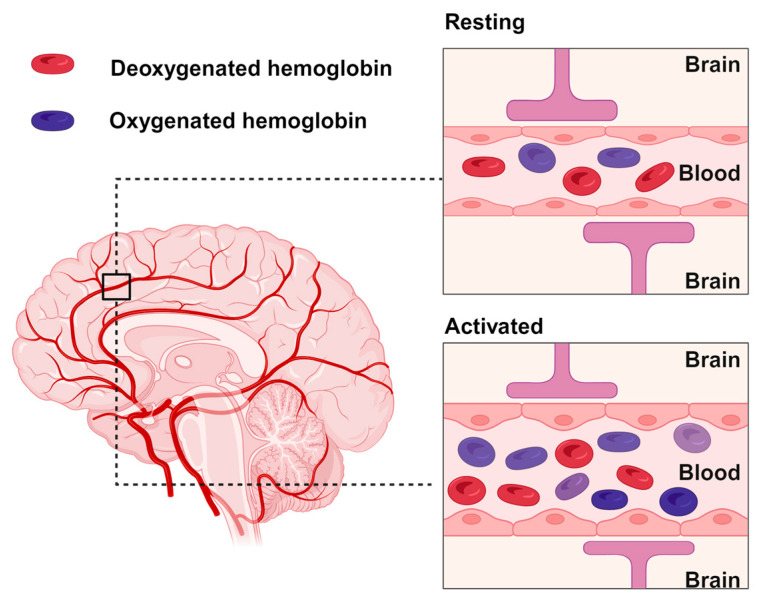
Principles of the HRF.

Blood oxygenation level dependent (BOLD) functional MRI (fMRI) sequences are by far the most widely applied scanning method for studying functional dynamics of the human brain in vivo [4]. Based on a cascade of cellular and chemical events that link activity to local changes of oxyhemoglobin and deoxyhemoglobin in the blood supply in the haemodynamic response function (HRF) BOLD fMRI provides an indirect measure of brain activity [5,6]. The hemodynamic response makes local blood vessels dilate, and more oxygenated blood flows to the active area. Oxygenated haemoglobin is diamagnetic (repelled by a magnetic field), while deoxygenated haemoglobin is paramagnetic (attracted to a magnetic field) [5] Changes in the balance between these forms of haemoglobin create detectable MRI signal variations, as illustrated in Figure 1. Notably the fMRI-BOLD approach specifically measures the change in signal, which fades out when new blood vessel composition has occurred. Compared to PET fMRI has a high temporal resolution in the order of seconds and enables implementation of dynamic measurements and multiple responses within a single scan [7,8]. However, BOLD fMRI is not a direct quantitative metric measurement of neural activity as a measure of brain function [4].

**Figure 2:**
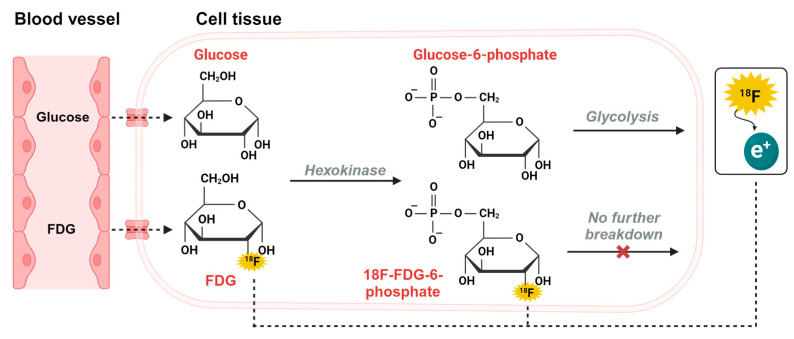
Principles of FDG-PET.

Technological advances in PET modality have yielded significantly better resolutions both spatially and temporally in PET scans and PET is now a technological candidate that can support the concept of functional, direct and quantitative dynamic scans. PET imaging is based on the principles of radioactivity, which involves injecting a radioactive tracer into the bloodstream. The advantages of PET imaging lies in the detail of the radiotracers, which can be designed to target specific biological processes or molecules [9]. Brain PET imaging applies especially well to these metabolic and biochemical processes, which gives insights into glucose metabolic activity in the cells and neurotransmitter receptor density. The widely available radiotracer 2-[^18^F]-flouro-2-deoxy-D-glucose (FDG) radiotracer, taken up as glucose in the hexokinase mediated reaction of cellular energy production, has long been established as a direct surrogate measure of glucose metabolism and extensively applied in static PET imaging in clinical cancer applications throughout the world [10]. FDG-PET studies examining glucose metabolism as a measure of dynamic neural activity provide an option for studying the brain on a functional level, as the brain uses about 25 percent of the body’s total glucose demand and 20% of the body’s oxygen consumption, illustrated in Figure 2 [11,12].

As an extension to the static PET imaging traditionally applied in clinical applications, functional PET (fPET) adds dynamics to a PET scan session by introducing paradigms during tracer uptake and hence change equilibria much akin to fMRI, constituting a potential for the future of functional brain scans related to task evoked neural activation. Early attempts of functional FDG (fFDG) scans for studying the human brain during both rest and task performance were often very time consuming and required arterial blood sampling during scans [13,14], later replaced by venous blood [15]. In recent years, various forms of fFDG for quantitative cerebral metabolic studies have emerged, suggesting and testing a variation of experimental designs, setups and analysis methods [3]. The main issues in these recent attempts have been to produce an applicable and reliable method for the implementation of fFDG scans concerning multiple parameters. These include the design of the activation paradigm, tracer administration setup, blood sampling and not least the choice of modelling in the analysis.

In general, all studies of task-related functional brain scans require a resting-state acquisition to establish the baseline condition to be compared to the activation condition. Studies performed on a combined PET/MRI scanner enabling both PET and MRI acquisition in a single scan provide the possibility to investigate structural, functional, and molecular biomarkers acquired in the same session, minimizing the chance of misalignment in data analysis, intra-subject variability due to physiological changes between scan sessions and reduces total scan time. Hence, numerous experimental design formats are currently being investigated in relation to functional fFDG scans [10,16].

**Figure 3:**
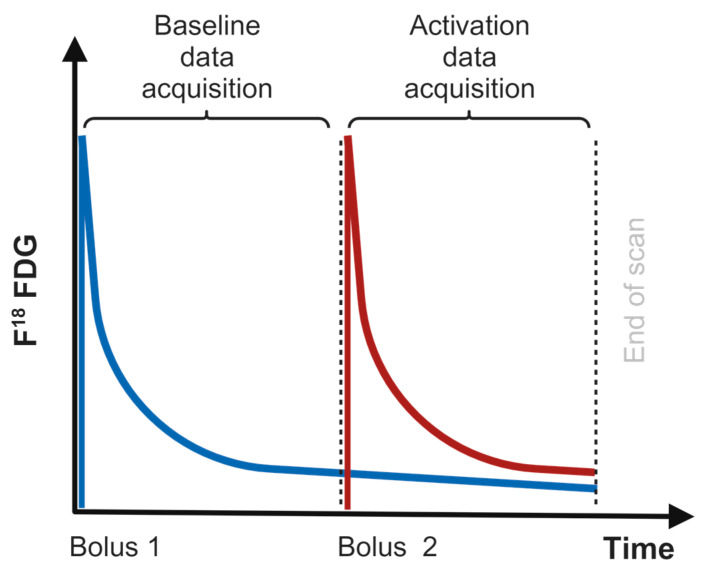
Principles of a double boli FDG tracer administration. Blue: First bolus. Red: Second bolus.

In this study, we apply a novel version of a two-compartment kinetic method for quantification of neural activity in a double boli tracer administration stand-alone PET design to detect changes due to task evoked activation [17]. In this double boli set-up, an injection with FDG is given twice over the course of the scan illustrated in Figure 3. The first injection is administrated simultaneously with the initiation of the PET scan acquisition. This marks the beginning of the baseline period. The second injection is administrated in the same way at the exact beginning of the activation period. The double boli tracer administration setup provides a favourable high FDG tissue response and accommodate a high rate of free tracer in both the baseline and activation phase. Furthermore, the double boli constructs a setup with similar FDG tracer conditions both at the baseline and activation phases. The compartmental analysis, in contract to the simplified Patlak approach, obviates the necessity for a stable equilibrium, consequently diminishing the temporal duration necessary for the determination of model parameters within the analytical framework [18,19,20]. Thereby reducing the time span needed for estimation of the model parameters in the analysis.

To support the fFDG scans, we applied the high-resolution T1-weighted MRI (T1) acquisition and fMRI-BOLD acquisition in the same scanning session as the fFDG-PET protocol. The structural T1 image served as a co-registration and common brain reference of the intra-subject PET and fMRI analyses and the fMRI as verification of origin of signal in subject space.

### 1.3. Activation Paradigm

The overall aim of the study is to create the basis of a methodology, which in future scenarios enables a straightforward and easy implementable tool for quantification of neural activity. With this approach we wish to create a basis for future studies and understanding of the pathology of disorders such as stress, depression, and anxiety. We believe functional scans are superior and necessary in future approaches in neuroimaging of the neurobiology of disorders that do not cause immediate neurodegeneration and structural changes in the brain. We used the finger-tapping activation paradigm, applied in neuroimaging as a mean for studying neural activity in the M1 area of the motor cortex [21].

## 2. Materials and Methods

All subjects were scanned on a 3.0T GE Healthcare SIGNA^TM^ PET/MR featuring simultaneous time of flight (TOF) PET imaging and a whole body 3.0T MRI scanner using a GE HeadNeckSpine 16 channel coil.

### 2.1. Participants

Five healthy subjects three female, two male, three right- and two left-handed, median age 42 years, height 173 cm, weight 85 kg, average blood glucose 5.44 ± 0.86 mmol/L were included.

The study was approved by the Ethics Committee of The Region of Southern Denmark Project-ID: S-20170086 and procedures were carried out in accordance with the Declaration of Helsinki. Written consent was acquired from all subjects before participating in the study procedure.

Participants were fasting for at least 5 h before FDG-tracer administration to allow optimal cerebral FDG uptake not to be influenced by increased blood glucose levels [22]. In the preparation routine a peripheral venous catheter was inserted in the hand and blood glucose levels measured. Participants were positioned supine in the scanner, placed in the head coil and fixed with foam pads to minimize head movement during scan.

### 2.2. Activation Paradigm

**Figure 4:**
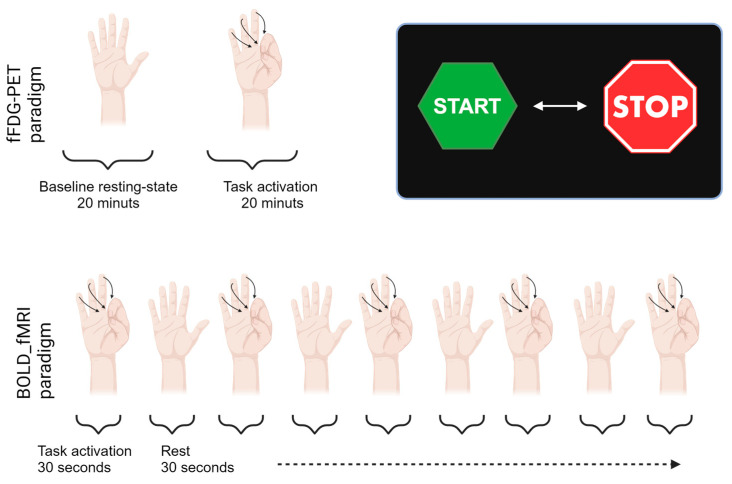
Finger-tapping paradigm.

In the finger-tapping task the participants tapped their fingers to the thumb on their right hand at a steady pace of about 1 Hz [23]. Visual instructions for commencement and completion of the finger-tapping paradigm were projected on a Nordic Neuro Lab system screen viewed via a mirror placed on the head coil above the eyes. The fFDG paradigm lasted for 40 min. Initially, a baseline was established for the first 20 min after injection, where participants rested with no form of external stimuli constituting our baseline resting-state acquisition. Hereafter, the finger-tapping paradigm was initiated and continued throughout the last 20 min of the dynamic PET scan. Following a short break after the 20 min continuous PET finger-tapping, a fMRI BOLD activation of 4 min and 30 s was initiated. The fMRI paradigm was built up by 30 s alternating blocks of START and STOP finger-tapping activation indicated by a presentation of visual START or STOP signs on the screen. The composition of the respective activation paradigms are illustrated in Figure 4.

### 2.3. FDG Administration and Data Acquisition

The [^18^F]FDG tracer was produced according to GMP specifications. A 40 min PET list-mode acquisition was recorded, and with a short delay, the first bolus injection of approximately 150 MBq [^18^F]FDG was administrated by a MRidium 3860+ MRI IV Infusion pump. The tracer was dissolved in 4.5 mL saline and injected at a rate of 600 mL/h, lasting 24 s in total. After 20 min scan time, and at the beginning of the activation session, an identical bolus was administrated to increase the amount of free tracer accessible to the task induced changes in glucose metabolism.

### 2.4. Image Processing and Data Analysis

A T1-weighted MRI scan (MRI T1: Sag T1W 3D FSPGR BRAVO (TE 2.3, TR 6.44 ms, voxel size 0.5 × 0.5 × 0.5 mm^3^) was acquired for all subjects to serve as the mutual structural original image for the aggregation of the FDG-PET analysis results and the fMRI analysis results. Intra-subject analysis images were analysed entirely in native space without normalization.

The image data for both modalities were analysed separately, and then compared. All subjects were analysed separately on an intra-subject level exclusively. The functional scans were quantified in two steps regarding the FDG-PET data for the baseline acquisition and the activation task acquisition, and the fMRI BOLD data analysis following in a separate analysis. Finally, the fFDG and the fMRI results were compared on spatial peak seed regions. The fFDG data were processed in Siemens Inveon Research Workplace, Advanced Normalization Tools (ANTs) (BSD License, NITRC, https://www.nitrc.org/projects/ants (accessed on 3 October 2023)) ANTs and custom-made Python code. fMRI data were processed in SPM12 (https://www.fil.ion.ucl.ac.uk/spm/software/spm12/ (accessed on 3 October 2023)). Single subject fFDG and fMRI were then warped to standard space and processed at group level in SPM12 for visual comparison of the mean fFDG and fMRI results in peak seed region.

### 2.5. fMRI

The fMRI BOLD data (EPI fMRI-TE 30, TR 2000 ms, native voxel size 3.75 × 3.75 × 4.0 mm) were processed in SPM12 fMRI module with default parameters maintained unless otherwise specified, including estimating options smoothing kernel (FWHM) 5, register to first, interpolation 2nd Degree B-spline, no wrap, and reslice options All Images + Mean image, interpolation 4th Degree B-Spline and Mask images. The T1 images were skull-stripped with FSL BET (Brain Extraction Tool) with T2-weighted FLAR acquisition as mask [24]. The realigned images were coregistered to the skull-stripped T1-weigthed structural image by applying estimation only. No further smoothing was applied to the single subject fMRI data. A grey matter, white matter and cerebrospinal fluid mask were created, to exclude non-brain matter from the analysis.

For group level analysis the single subject fMRI data was first coregistered to the individual T1 structural images and resliced to voxel size [2 2 2], smoothed by a Gaussian kernel of [8 8 8] and normalized to Montreal Neurological Institute coordinate system (MNI) standard space with estimate and write. In the group level, the stats paradigm parameters were set identical to those applied to the single subject analysis, However, with no explicit mask.

### 2.6. PET

The PET listmode files (Axial PET field-of-view 25 cm) were reconstructed with the GE Healthcare Q.Clear algorithm with a β-value of 500. Attenuation correction maps were supplied using GE zero-echo-time (ZTE)-based attenuation correction for integrated PET/MRI brain imaging. In the fFDG data, the first two minutes were framed in 8 × 15 s, following 9 frames of 2 min in the baseline acquisition. This framing was repeated for the remaining 20 min of scan time during activation. The data were reconstructed with an anisotropic voxel size of 2.34 × 2.34 × 2.78 mm^3^ into a volume of 89 slices of 128 × 128 pixels. Intraframe PET motion corrections for the dynamic series were applied by rigid registration in ANTs using the final frame as reference.

**Figure 5:**
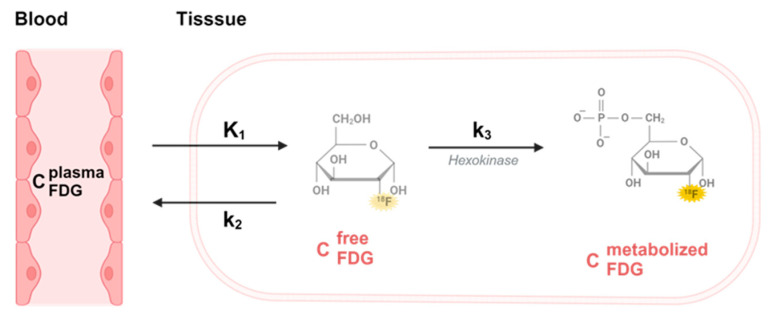
Two-tissue compartment model for irreversible tracers illustrating the free and metabolized compartments and the kinetics of the rate constant K_1_, k_2_ and k_3_ kinetics.

FDG data was modelled by the approach initially applied by Murase et al. in a double boli administration application [17]. We evaluated the FDG tissue uptake using a two-tissue compartment model for irreversible tracers with a free (**C_f_**) and a metabolized (trapped) compartment (**C_m_**). $K_{1}$ is the unidirectional blood-to-tissue clearance, $k_{2}$ is the rate constant for clearance of FDG from tissue to blood and $k_{3}$ is the rate constant for FDG trapping illustrated in Figure 5. 

Input function for modelling was defined by an image-derived input curve [25]. The model forms the foundation for determining the relationship between the dynamic measure of change in FDG metabolism and, hence, represent the cerebral metabolic rate of glucose (CMRglu) in the baseline and during the finger-tapping activation.

The underlying differential equation that describes the free compartment function $C_{f}$ is:

(1) $$\frac{{dC}_{f}}{dt}=K_{1}C_{a}\left(t\right)-\left(k_{2}+k_{3}\right)C_{f}\left(t\right)$$

And the equation describing the metabolized compartment function $C_{m}$ is given by:

(2) $$\frac{{dC}_{m}}{dt}=k_{3}C_{f}\left(t\right)$$

where $C_{a}$(t) is the (arterial) blood FDG concentration at time t. 

The PET measured total FDG activity concentration $C_{T}$ is the sum of both compartments with an additional small contribution from the vascular blood FDG activity component, as follows:

(3) $$C_{T}\left(t\right)=C_{f}\left(t\right)+C_{m}\left(t\right)+V_{0}C_{a}\left(t\right)$$

where $V_{0}$ is the vascular blood volume.

The parameter of interest in this study is the net uptake rate $K_{i}$ of FDG, which is determined as:

(4) $$K_{i}=\frac{K_{1}k_{3}}{k_{2}+k_{3}}$$

The cerebral metabolic rate for glucose ${CMR}_{glucose}$ is directly proportion to $K_{i}$ through the equation:

$${CMR}_{glucose}=K_{i}\frac{C_{Pglucose}}{LC}$$

where $C_{Pglucose}$ is the plasma glucose concentration and **LC** is the lumped constant connecting the FDG rate constant $K_{i}$ to the glucose rate constant [26]. A relative change in $K_{i}$ is equal to the relative change in the glucose metabolism, assuming a constant plasma glucose concentration between the two sessions.

To calculate the change in glucose metabolism between baseline and activation the PET acquisition analysis was divided into two consecutive sessions, with initial bolus injection at the beginning (**t** = 0 min) and the second bolus injection at beginning of the activation session (**t_2_** = 20 min).

Quantification with double boli.

The solution to the baseline session **t** < 20 min is given by:

(5) $$C_{f,baseline}\left(t\right)=K_{1,1}\int_{0}^{t}e^{-\left(k_{2,1}+k_{3,1}\right)\left(t-u\right)}C_{a}\left(u\right)du$$

(6) $$C_{m,baseline}\left(t\right)=\frac{K_{1,1}k_{3,1}}{k_{2,1}+k_{3,1}}\int_{0}^{t}\left({1-e}^{-\left(k_{2,1}+k_{3,1}\right)\left(t-u\right)}\right){C_{a}\left(u\right)du}_{}$$

where $K_{1,1}{,k}_{2,1}$ and $k_{3,1}$ denotes the baseline model parameters.

While the solution for the activation session **t** > 20 min has an extra term due to initial boundary conditions originating from the previous baseline session:

(7) $$C_{f,activation}\left(t\right)=K_{1,2}\int_{t_{2}}^{t}e^{-\left(k_{2,2}+k_{3,2}\right)\left(t-u\right)}C_{a}\left(u\right)du+e^{-\left(k_{2,2}+k_{3,2}\right)\left(t-t_{2}\right)}C_{f,baseline}\left(t_{2}\right)$$

(8) $$C_{m,activation}\left(t\right)=\frac{K_{1,2}k_{3,2}}{k_{2,2}+k_{3,2}}\int_{t_{2}}^{t}\left({1-e}^{-\left(k_{2,2}+k_{3,2}\right)\left(t-u\right)}\right)C_{a}\left(u\right)du+C_{m,baseline}\left(t_{2}\right)$$

where $K_{1,2}{,k}_{2,2}$ and $k_{3,2}$ denotes session 2 (activation) model parameters. $t_{2}$ is the time for beginning of session 2 ($t_{2}$ = 20 min).

To obtain the blood input function $C_{a}$, we used a non-invasive PET image-derived method. From the first minute of the acquisition, we obtained a well-defined image of the blood vessels below the brain. Using a simple threshold technique, we segmented the blood vessels. To reduce the sensitivity from potential patient movement and partial volume effect (**PVE**), we dilated the volume of interest (**VOI_vessel_**) with 6 mm in all directions. An area in the neck region without any notable blood vessels constitutes the reference volume (**VOI_bkg_**) for the background correction to the image derived input function. The image derived blood input function is calculated as:

(9) $$C_{a}\left(t\right)=\left({VOI}_{vessel}-{VOI}_{bkg}\right)PVC$$

where **PVC** is a partial volume correction constant that corrects the volume to the true vessel volume. From the differential Equation (1) above, it is seen that the **PVC** constant is a simple multiplication factor just like the $K_{1}$ parameter, which implies that $K_{i}$ becomes proportional to **PVC** and hence the relative change of $K_{i}$ between baseline and activation sessions is independent of the **PVC** constant.

### 2.7. Vascular Blood Volume

The short time frames and the low injected activity implies a rather high statistical noise level in the raw data. To reduce the noise, we applied an 8 mm FWHM Gaussian spatial 3-dimensional smoothing filter on all individual frames.

The model parameters $K_{1,1}{,k}_{2,1}$ and $k_{3,1}$ are calculated voxelwise. On a voxel scale the vascular blood volume $V_{0}$ fluctuates hugely and depends on the distribution of larger vessels in the brain.

Equation (3) can be re-arranged as $V_{0}=\frac{{C_{T}(t)-C}_{f}\left(t\right)-C_{m}\left(t\right)}{C_{a}\left(t\right)}$.

However, the effect of $V_{0}$ on the time activity curves (TAC) $C_{T}\left(t\right)$ is predominately in the early vascular phase. Estimating the $C_{f}$ and $C_{m}$ from data in the time span from 4 min after start of acquisition until end of baseline session, we can estimate parameters $K_{1,1}{,k}_{2,1}$ and $k_{3,1}$ with minor influence from the vascular blood volume $V_{0}$. Using these parameters in the early phase dominated by the vascular component we can calculate $V_{0}$ from the above equation as the average value from 15 s prior and until the vascular peak in $C_{b}$. $V_{0}$ was calculated using the smoothed data.

To reduce the possibility of the fitting algorithm getting trapped in local minima produced by noise we initiate the parameter estimation of $K_{1,1}{,k}_{2,1}$ and $k_{3,1}$ using the smoothed baseline data. Next, we fitted the raw data with constraints that the new estimated parameters should lie in the interval 0.25 to 4 times the initial estimate and kept $k_{3,1}$ constant. Finally, we estimated the parameters from the activation session $K_{1,2}{,k}_{2,2}$ and set $k_{3,2}$ equal to $k_{3,1}$. Again, to reduce the possibility that the fitting algorithm gets trapped in local minima the activation parameters was constrained to lie in the interval from 0.5 to 2.5 times the baseline parameters. The parameter constrain intervals were chosen based on the need for narrow limits due to noise. However, the limits are large enough to allow for the relevant physiological changes from baseline session to the activation session.

The final parametric image was calculated as $∆K_{i}=100\%\frac{K_{i.2}-K_{i,1}}{K_{i,2}}$. All parameter estimations were done using Lmfit, a least-square Levenberg–Marquardt algorithm implemented in Python (v1.0.3, GitHub, https://lmfit.github.io/lmfit-py/ (accessed on 3 October 2023)).

A visualisation of the seed peak regions of the fMRI and fFDG results were projected on the single subject T1 structural image and at group level the MNI standard T1 structural brain template using FSLeyes version 1.3.0 software (https://git.fmrib.ox.ac.uk/fsl/fsleyes/fsleyes/ (accessed on 3 October 2023)). Peak values were extracted for all five participants and the group mean level.

## 3. Results

**Figure 6:**
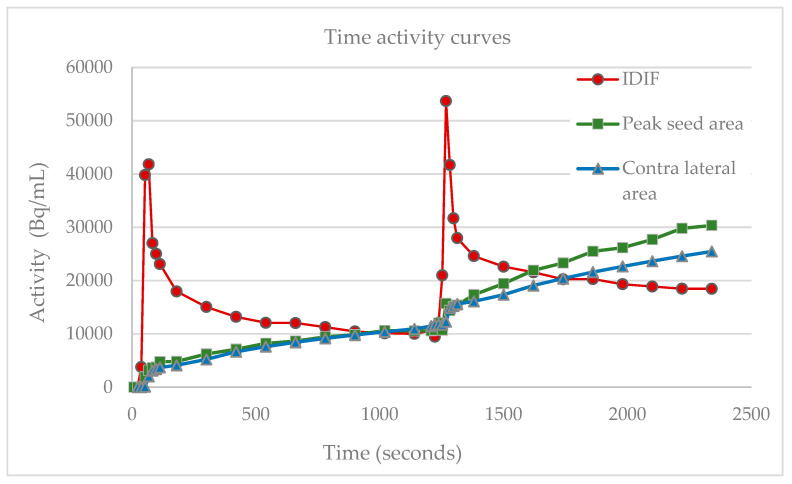
Double boli time activity curves.

The adapted two-tissue compartment model for quantification of task-evoked neural activity provided a consistent solution to determine the relative change in $K_{i}$, representing the relative change in glucose metabolism, independent of the PVC constant and with a calculated V_0_ at the voxel level shown in Figure 6.

Change in the slope of the motor cortex M1 measured during baseline and activation is equal to changes in net uptake rate constant K_i_ represented by FGD-uptake, illustrated with the contralateral reference region of the peak effect and blood component curves. The abbreviations used are as follows: IDIF, image derived input function; Bq, Bequerel; mL, milliliters.

Applying the two-tissue compartment model to the double boli scanner paradigm design the relative changes in **K_i_** in the M1 peak seed area increased with 36.3% to 87.9% for the finger-tapping task at single subject level. Likewise, the single subject fMRI paradigm analysis showed a statistically significant task specific activation in the M1 area measured by the BOLD signal with peak t-values from 15.2–27.6, average 21.7. Single-subject peak values for the relative rise in FDG uptake and fMRI peak T-values are listed in Table 1.

**Figure 7:**
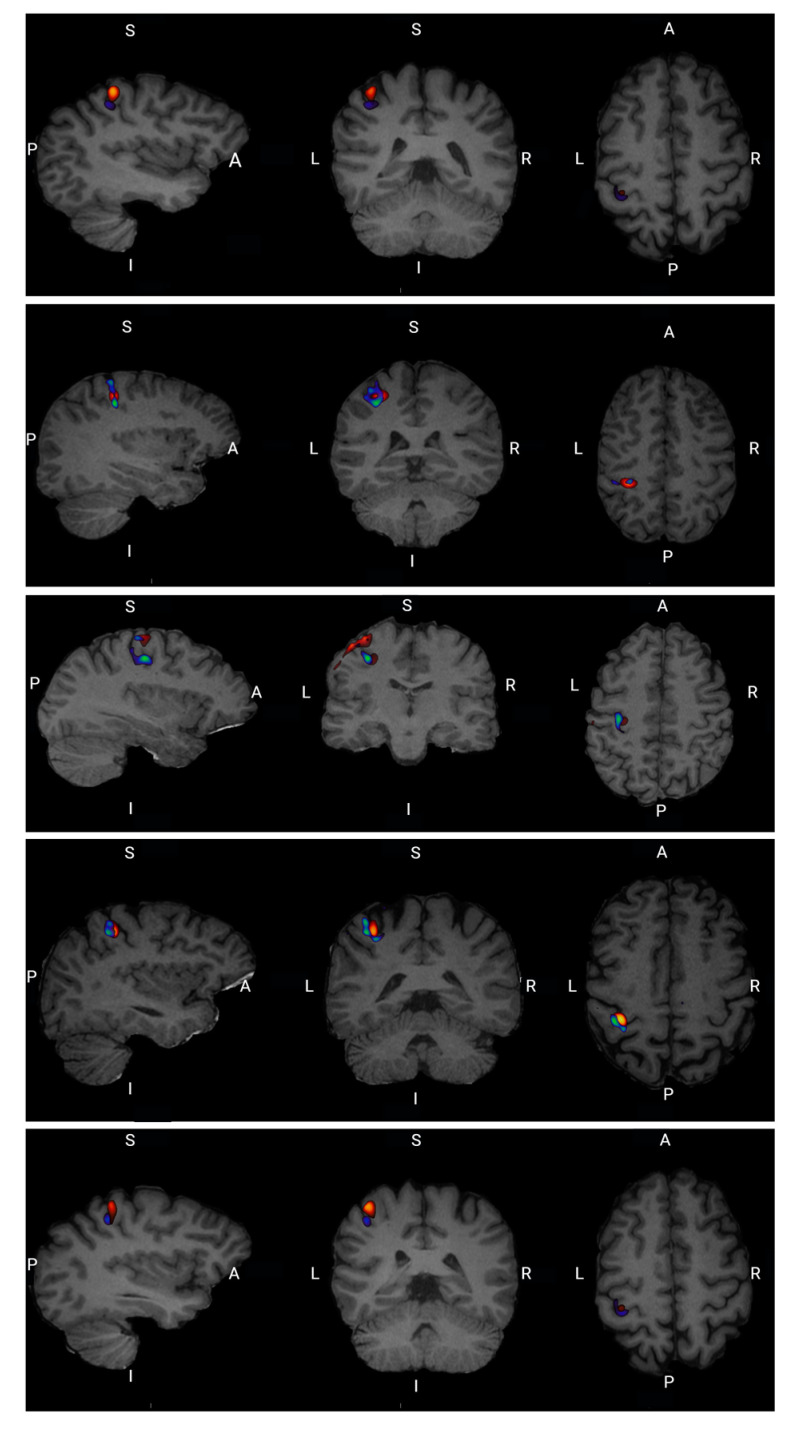
Single subject 1–5 functional FDG and fMRi images merged and superimposed on subject T1 structural MRI image. A: anterior, P: posterior, S: superior, I: inferior, L: left, R: right.

For all five participants there is a visual overlap between task-related neural activation in FDG uptake and the fMRI BOLD signal at the single-subject level as well as at the group level illustrated in Figure 7.

**Figure 8:**
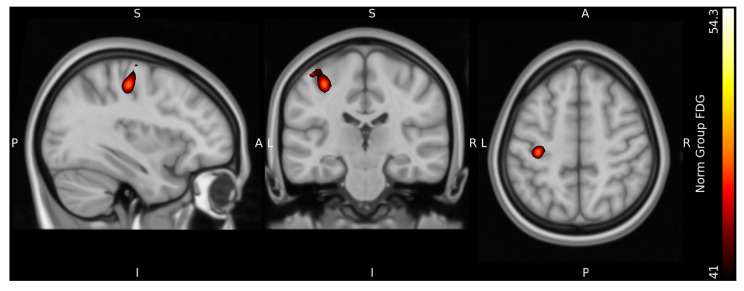
Group-level functional FDG images normalized to Montreal Neurological Institute (MNI) standard space superimposed on the MNI avg152T1 template. A: anterior, P: posterior, S: superior, I: inferior, L: left, R: right.

**Figure 9:**
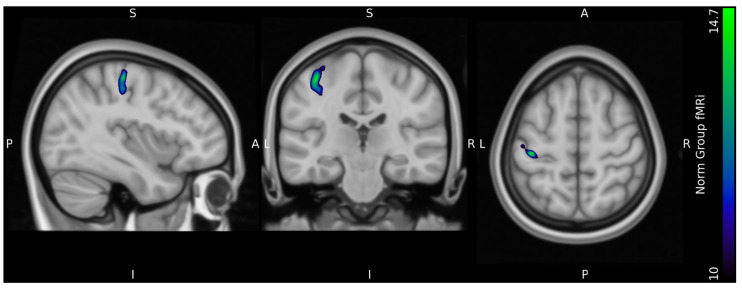
Group-level fMRI images normalized to Montreal Neurological Institute (MNI) standard space superimposed on the MNI avg152T1 template. AA: anterior, P: posterior, S: superior, I: inferior, L: left, R: right.

**Figure 10:**
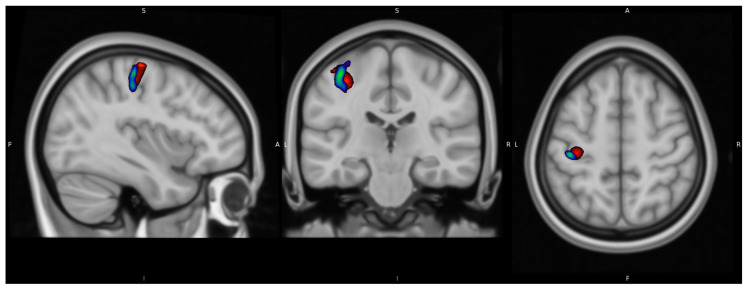
Normalized merged group fFDG and fMRi image superimposed on Montreal Neurological Institute (MNI) standard space on the MNI avg152T1 template. A: anterior, P: posterior, S: superior, I: inferior, L: left, R: right.

Figure 8 and Figure 9 show the results at the group level normalized to MNI standard space. At the group level, the resulting peak seed region of both signals were located in the in the dorsal portion of the frontal lobe at the precentral gyrus in Brodmann area 4, in accordance with the M1 motor cortex region of the left cerebrum. Figure 10 illustrates a merged image of the fFDG and fMRI peak areas, with the same illustrative thresholds as in Figure 4 and Figure 5. Video S1 illustrates both the fFDG and fMRI peak seed regions in T1 MNI standard space.

## 4. Discussion

In the interest of improving facilities to understand the pathology of disorders such as stress, depression, anxiety and other psychiatric diseases with fluctuating symptoms but no immediate structural changes in the brain, this study applied a novel implementation of a double boli administration of [^18^F]FDG to investigate the change in FDG uptake between baseline and activation PET acquisition as a measure of glucose consumption evoked by neural activity in a single-scanning session at the single-subject level. The finger-tapping activation paradigm was redesigned for a PET-standalone setup, followed by a conventional fMRI version in the same scanning session.

In the analysis of the fFDG data, we concentrated on the measurement of relative changes in FDG uptake, as a measure of glucose metabolism in accordance with previous research [27]. As blood input function we have used an image derived input function (IDIF), which, in the case of relative estimation of changes kinetic parameters, is applicable assuming no non-linear bias between the true input function and the selected IDIF. Using this approach our results demonstrated that the double boli approach with [^18^F]FDG and an image-derived input function (IDIF) enables quantification of relative changes in K_i_, with task-specific changes in glucose metabolism increasing by 36.3–87.9% (max) locally in the brain, on an intra-subject level. The image analysis of the corresponding fMRI paradigm, also based on the finger-tapping paradigm, confirmed the spatial peak area of the fFDG results in the M1 motor cortex region of the human brain. Visual inspection showed minor deviation in the extension of the spatial location of neural activity between the fFDG-PET and fMRI-BOLD paradigm.

Our main objectives of the implementation of the two-tissue compartment kinetic model in our analysis was to apply a method that incorporates the entire dataset by estimating changes in rate constants between baseline and activation, without the need of blood sampling.

The discussion of the optimal tracer administration protocol in the form of bolus vs. constant infusion, as well as applying an initial bolus injections followed by constant infusion of FDG, is ongoing [28]. In recent years, constant infusion has been praised [10,16] as a superior method, creating greater sensitivity over a single administrated bolus, as it offers a constant supply of FDG during baseline and activation sessions. Whereas a single bolus will truly offer reduced quantities of FDG at the time of the activation session after the baseline session, a double bolus fFDG method, which we applied, provides high amounts of tissue-available FDG activity, yielding a strong quantitative measure of cerebral metabolic rate in the baseline as well as the activation session. This enables a powerful approach to characterize functional metabolic responses to stimuli that are presumed to sustain a constant state, including visual, auditory or cognitive tasks, as well as drug administration [29,30,31,32]. In other studies, the well-known fMRI analysis pipeline has served as inspiration to the fFDG scanning paradigm expressed in a combined simultaneously PET/fMRI-BOLD acquisition block design in a single scan with both baseline and activation [28]. 

The variation of tracer administration and scanning paradigm protocols in the fFDG task evoked change in FDG uptake has resulted in various forms of analysis being applied. As the design of the traces administration activation paradigm to some extent dictates the analysis model and vice versa, no gold-standard experimental design setup has been presented. The variation include studies that applies fMRI-BOLD features from the fixed method of the general linear model (GLM), with normalization of the material, as well as the graphical approach by the Patlak model based on the assumption of a steady-state in FDG between compartments and independent component analysis (ICA) [33]. In the same manner, the blood sampling to support the input function varies from arterial to venous, both automatically and manually acquired [10]. However, the incorporation of the GLM line introduces indirect components from the HRF in the otherwise quantifiable PET analysis of glucose consumption. The proposed method using a full two-tissue compartment model we process the data of the entire TAC, hence the parameter estimation uses all available time points in opposite to the simple Patlak approach that only uses data from later time points when a quasi-equilibrium between the free and metabolized compartment are reached. Also, the Patlak model does not account for the spatially variable vascular component.

In general, the implementation of the widely available glucose analogue radiotracer FDG to investigate quantifiable neural activity at rest and under activation connected to a specific task related brain function in health and mental disorders holds a vast potential. One of the advantages of our method is, firstly, an easily implementable setup in terms of the double boli tracer administration, without the need for invasive blood sampling, arterial or venous, to support the input function for individual quantification [15]. An IDIF may have quantitative bias compared to measured arterial blood samples and, hence, produce a bias in our measurements of glucose metabolic rate K_i_. However, by adopting a relative change approach, the IDIF approach remains accurate in absence of non-linear bias effects. Even though our study is based on a small sample size, as a proof-of-concept, our results shows that our novel approach of the double-boli application of the two-compartmental model is consistent across all five single subjects included, as well as at the subject level and comparable to the similar studies [10].

As for the implementation of our adapted two-tissue compartment model in this study, the calculated values related to the change in FDG uptake, are somewhat higher compared to other studies addressing functional FDG scans with task evoked neural activation by other methods. As an example, the results in the other studies range from approximately 20% to 30% [10,16] in the peak seed region, compared to our resulting values of 36.3% to 87.9%. These variances in the values could be due to different activation paradigms, the method of addressing the analysis, and whether data is normalized to standard space during analysis. However, the discrepancy could also be associated with an inaccuracy in the input function in our method, rather than an actual difference in the measures of the physiological signal linked to the scans.

Related Associated Anatomical minute differences in the peak area of the fFDG signal and the fMRI can be visually observed, as it has been in other studies [15]. The deviations in the peak seed regions of the fFDG results and the fMRI results holds the potential to study the physiological relation between glucose consumption and the flow of changes in deoxyhemoglobin in the brain determined by localized changes in blood flow and blood oxygenation in the neurovascular coupling with the PET/MRI modality. This aspect has been observed in other human studies, as well as in rats. It is suggested that FDG uptake could be linked to an additional physiological process or secondary task-related regions not detected by fMRI-BOLD [28,34]. Also the characteristics of the signal-to-noise ratio differs between the two methods, which not only affects the statistical power of the methods to detect effects, but also the proportion of voxels/clusters that exceed statistical threshold [15]. Thus, the relation between the consumption of glucose and oxygen in the brain is a rising area of interest related to understanding abnormal brain activity and disease [35,36]. 

In a future clinical aspect, our method is simple to adapt and implement as a PET stand-alone setup or accompanied by a fMRI sequence, in sites where scanners featuring the only PET scanner facilities or the double PET/MRI modality. The setup with a double boli administration is easily executed and can even be carried out manually. The purely imaging-based approach without the need of blood sampling made possible by the IDIF, further supports this setup. The two-tissue kinetic compartment analysis is compiled in a Python script. New whole-body approximated PET-scanners further support much more sensitivity, hence the absolute and robust quantification of data and better delineation of small vessel and less noisy imaging in general. The two-tissue compartment method uses the full data range and in theory has no lower time limit, relying only on image quality and the derived TAC’s. Our fFDG model improves the overall future clinical aspects within mental health in the study of neural activity related to changes in function and fluctuating symptoms in brain disorders, also including the implications related to brain plasticity and compensations related to lesions and trauma, etc.

One noticeable limitation of this study is the long scan times of the PET stand-alone paradigm in both the baseline period, but in particular the activation period, which could probably cause some fatigue of the hands and fingers of the test subjects and/or habituation effects in neural activation [37]. We also assume that the rate constants are non-variable during both baseline and activation. The method should be tested with shorter time paradigms. The limiting factor is only image quality. With an increase in scanner sensitivity, we could decrease the scan time proportionally. Furthermore, we are now applying the method in studies of dynamic changes of neural activity measured by dynamic changes in the glucose metabolism in activation paradigms in relation to cognitive functions, including higher-order functions such as attention, memory and executive functions.

## 5. Conclusions

In conclusion, this study implemented a novel application of a double boli administration and a two-tissue compartment model for the analysis of the measurement of FDG uptake induced by task-evoked neural activation with a finger-tapping paradigm and a fMRI acquisition for comparison of peak seed region in the human brain. The study confirmed that it is indeed possible to enable quantitative brain imaging on a functional level, with task-specific changes in glucose utilization, with an accuracy that supports future research in entirely new knowledge of brain functions in mental disorders and health and pharmacological treatment mechanisms.

## 6. Illustrations

All graphical illustrations were created by Saga Steinmann Madsen licensed in BioRender.com/FSLeyes.

## Figures and Tables

**Table 1:** Relative changes in fFDG Ki and fMRI peak T-values.

Subject	Relative Peak Rise in FDG Uptake	fMRI Peak T-Values
1	87.9	27.6
2	67.7	18.3
3	71.7	23.8
4	46.6	15.2
5	36.3	23.4
Mean	62.0	21.7
Normalized mean	54.3	14.7

## Data Availability

Data can be shared upon request.

## Supplementary Materials

The following supporting information can be downloaded at: https://www.mdpi.com/article/10.3390/diagnostics13193121/s1. Video S1_GIF_fFDG_fMRI: Functional FDG (fFDG) and fMRI peak seed area in T1 Montreal Neurological Institute coordinate system standard space. Orange; FDG. Blue; fMRI.

## References

[1] World Health Organization (WHO) (2022). Mental Disorders: Key Facts. https://www.who.int/news-room/fact-sheets/detail/mental-disorders.

[2] Feigin V.L., Nichols E., Alam T., Bannick M.S., Beghi E., Blake N., Culpepper W.J., Dorsey E.R., Elbaz A., Ellenbogen R.G. (2019). Global, regional, and national burden of neurological disorders, 1990–2016: A systematic analysis for the Global Burden of Disease Study 2016. Lancet Neurol..

[3] Chiaravalloti A., Micarelli A., Ricci M., Pagani M., Ciccariello G., Bruno E., Alessandrini M., Schillaci O. (2019). Evaluation of Task-Related Brain Activity: Is There a Role for (18)F FDG-PET Imaging?. Biomed. Res. Int..

[4] Hill P.F., Seger S.E., Yoo H.B., King D.R., Wang D.X., Lega B.C., Rugg M.D. (2021). Distinct neurophysiological correlates of the fMRI BOLD signal in the hippocampus and neocortex. J. Neurosci..

[5] Norris D.G. (2006). Principles of magnetic resonance assessment of brain function. J. Magn. Reson. Imaging.

[6] DeYoe E.A., Raut R.V. (2014). Visual mapping using blood oxygen level dependent functional magnetic resonance imaging. Neuroimaging Clin. N. Am..

[7] Kwong K.K., Belliveau J.W., Chesler D.A., Goldberg I.E., Weisskoff R.M., Poncelet B.P., Kennedy D.N., Hoppel B.E., Cohen M.S., Turner R. (1992). Dynamic magnetic resonance imaging of human brain activity during primary sensory stimulation. Proc. Natl. Acad. Sci. USA.

[8] Ogawa S., Tank D.W., Menon R., Ellermann J.M., Kim S.G., Merkle H., Ugurbil K. (1992). Intrinsic signal changes accompanying sensory stimulation: Functional brain mapping with magnetic resonance imaging. Proc. Natl. Acad. Sci. USA.

[9] Morrocchi M., Guerra A.D. (2020). Positron Emission Tomography: Alive and kicking after more than 65 years on stage. J. Instrum..

[10] Villien M., Wey H.Y., Mandeville J.B., Catana C., Polimeni J.R., Sander C.Y., Zürcher N.R., Chonde D.B., Fowler J.S., Rosen B.R. (2014). Dynamic functional imaging of brain glucose utilization using fPET-FDG. NeuroImage.

[11] Lundgaard I., Li B., Xie L., Kang H., Sanggaard S., Haswell J.D., Sun W., Goldman S., Blekot S., Nielsen M. (2015). Direct neuronal glucose uptake heralds activity-dependent increases in cerebral metabolism. Nat. Commun..

[12] Mergenthaler P., Lindauer U., Dienel G.A., Meisel A. (2013). Sugar for the brain: The role of glucose in physiological and pathological brain function. Trends Neurosci..

[13] Reivich M., Kuhl D., Wolf A., Greenberg J., Phelps M.A., Ido T., Casella V., Fowler J., Hoffman E., Alavi A. (1979). The [^18^F]fluorodeoxyglucose method for the measurement of local cerebral glucose utilization in man. Circ. Res..

[14] Phelps M.E., Huang S.C., Hoffman E.J., Selin C., Sokoloff L., Kuhl D.E. (1979). Tomographic measurement of local cerebral glucose metabolic rate in humans with (F-18)2-fluoro-2-deoxy-D-glucose: Validation of method. Ann. Neurol..

[15] Jamadar S.D., Ward P.G., Li S., Sforazzini F., Baran J., Chen Z., Egan G.F. (2019). Simultaneous task-based BOLD-fMRI and [18-F] FDG functional PET for measurement of neuronal metabolism in the human visual cortex. Neuroimage.

[16] Hahn A., Gryglewski G., Nics L., Hienert M., Rischka L., Vraka C., Sigurdardottir H., Vanicek T., James G.M., Seiger R. (2016). Quantification of Task-Specific Glucose Metabolism with Constant Infusion of 18F-FDG. J. Nucl. Med..

[17] Murase K., Kuwabara H., Yasuhara Y., Evans A.C., Gjedde A. (1996). Mapping of change in cerebral glucose utilization using fluorine-18 fluorodeoxyglucose double injection and the constrained weighted-integration method. IEEE Trans. Med. Imaging.

[18] Patlak C.S., Blasberg R.G. (1985). Graphical evaluation of blood-to-brain transfer constants from multiple-time uptake data. Generalizations. J. Cereb. Blood Flow Metab..

[19] Gjedde A. (1982). Calculation of cerebral glucose phosphorylation from brain uptake of glucose analogs in vivo: A re-examination. Brain Res..

[20] Ripp I., Cabello J., Yakushev I. (2019). Significant changes of regional cerebral FDG uptake during a steady state. J. Nucl. Med..

[21] Witt S.T., Laird A.R., Meyerand M.E. (2008). Functional neuroimaging correlates of finger-tapping task variations: An ALE meta-analysis. Neuroimage.

[22] Varrone A., Asenbaum S., Vander Borght T., Booij J., Nobili F., Någren K., Darcourt J., Kapucu Ö.L., Tatsch K., Bartenstein P. (2009). EANM procedure guidelines for PET brain imaging using [^18^F]FDG, version 2. Eur. J. Nucl. Med. Mol. Imaging.

[23] Rao S.M., Bandettini P.A., Binder J.R., Bobholz J.A., Hammeke T.A., Stein E.A., Hyde J.S. (1996). Relationship between finger movement rate and functional magnetic resonance signal change in human primary motor cortex. J. Cereb. Blood Flow Metab..

[24] Smith S.M. (2002). Fast robust automated brain extraction. Hum. Brain Mapp..

[25] Silvestri E., Volpi T., Bettinelli A., De Francisci M., Jones J., Corbetta M., Cecchin D., Bertoldo A. (2022). Image-derived Input Function in brain [(18)F]FDG PET data: Which alternatives to the carotid siphons?. Annu. Int. Conf. IEEE Eng. Med. Biol. Soc..

[26] Barrio J.R., Huang S.C., Satyamurthy N., Scafoglio C.S., Amy S.Y., Alavi A., Krohn K.A. (2020). Does 2-FDG PET Accurately Reflect Quantitative In Vivo Glucose Utilization?. J. Nucl. Med..

[27] Riedl V., Bienkowska K., Strobel C., Tahmasian M., Grimmer T., Förster S., Friston K.J., Sorg C., Drzezga A. (2014). Local activity determines functional connectivity in the resting human brain: A simultaneous FDG-PET/fMRI study. J. Neurosci..

[28] Rischka L., Gryglewski G., Pfaff S., Vanicek T., Hienert M., Klöbl M., Hartenbach M., Haug A., Wadsak W., Mitterhauser M. (2018). Reduced task durations in functional PET imaging with [(18)F]FDG approaching that of functional MRI. Neuroimage.

[29] Kushner M.J., Rosenquist A., Alavi A., Rosen M., Dann R., Fazekas F., Bosley T., Greenberg J., Reivich M. (1988). Cerebral metabolism and patterned visual stimulation: A positron emission tomographic study of the human visual cortex. Neurology.

[30] Pietrini P., Alexander G.E., Furey M.L., Dani A., Mentis M.J., Horwitz B., Guazzelli M., Schapiro M.B., Rapoport S.I. (2000). Cerebral metabolic response to passive audiovisual stimulation in patients with Alzheimer’s disease and healthy volunteers assessed by PET. J. Nucl. Med..

[31] Vlassenko A.G., Rundle M.M., Mintun M.A. (2006). Human brain glucose metabolism may evolve during activation: Findings from a modified FDG PET paradigm. Neuroimage.

[32] Yehuda R., Harvey P.D., Golier J.A., Newmark R.E., Bowie C.R., Wohltmann J.J., Grossman R.A., Schmeidler J., Hazlett E.A., Buchsbaum M.S. (2009). Changes in relative glucose metabolic rate following cortisol administration in aging veterans with posttraumatic stress disorder: An FDG-PET neuroimaging study. J. Neuropsychiatry Clin. Neurosci..

[33] Li S., Jamadar S.D., Ward P.G., Premaratne M., Egan G.F., Chen Z. (2020). Analysis of continuous infusion functional PET (fPET) in the human brain. Neuroimage.

[34] Wehrl H.F., Hossain M., Lankes K., Liu C.C., Bezrukov I., Martirosian P., Schick F., Reischl G., Pichler B.J. (2013). Simultaneous PET-MRI reveals brain function in activated and resting state on metabolic, hemodynamic and multiple temporal scales. Nat. Med..

[35] Ding C., Du W., Zhang Q., Wang L., Han Y., Jiang J. (2021). Coupling relationship between glucose and oxygen metabolisms to differentiate preclinical Alzheimer’s disease and normal individuals. Hum. Brain Mapp..

[36] Stiernman L.J., Grill F., Hahn A., Rischka L., Lanzenberger R., Panes Lundmark V., Riklund K., Axelsson J., Rieckmann A. (2021). Dissociations between glucose metabolism and blood oxygenation in the human default mode network revealed by simultaneous PET-fMRI. Proc. Natl. Acad. Sci. USA.

[37] Becerra L.R., Breiter H.C., Stojanovic M., Fishman S., Edwards A., Comite A.R., Gonzalez R.G., Borsook D. (1999). Human brain activation under controlled thermal stimulation and habituation to noxious heat: An fMRI study. Magn. Reson. Med..

